# Audiovisual decision making and sensory evidence weighting is not affected by impulsivity or impulsivity related behaviours in young adults

**DOI:** 10.1038/s41598-025-24668-3

**Published:** 2025-11-19

**Authors:** Rosanne R. M. Tuip, Jeannette A. M. Lorteije, Lucres M. C. Jansen, Tycho J. Dekkers, Filip Van Opstal

**Affiliations:** 1 Swammerdam Institute for Life Sciences, Center for Neuroscience, Faculty of Science, University of Amsterdam, Science Park 904, 1098 XH Amsterdam, The Netherlands; 2 Department of Psychology, Brain and Cognition, University of Amsterdam, Nieuwe Achtergracht 129, 1001 NK Amsterdam, The Netherlands; 3 Animal Welfare Body, Radboud University/UMC, Nijmegen, The Netherlands; 4 Department of Child and Adolescent Psychiatry & Psychosocial Care, Amsterdam UMC, Amsterdam, The Netherlands; 5 Accare Child Study Center, University of Amsterdam, Groningen, The Netherlands; 6 Department of Psychiatry, University Medical Center Groningen (UMCG), Groningen, Netherlands; 7 University of Amsterdam, Amsterdam, Netherlands; 8 Amsterdam Neuroscience, University of Amsterdam, Amsterdam, Netherlands

**Keywords:** Multisensory integration, Impulsivity, ADHD, DBD, Temporal integration, Perceptual decision-making, Psychology, Human behaviour

## Abstract

Multisensory integration enhances perceptual performance, often resulting in more accurate and efficient decision-making compared to unisensory processing. In contrast, impulsive behavior is associated with decreased decision accuracy and adverse outcomes. However, the influence of impulsivity on multisensory integration (MSI) remains poorly understood. This study investigated this relationship in a sample of 40 participants, including 10 individuals diagnosed with Attention-Deficit/Hyperactivity Disorder (ADHD) or Disruptive Behavior Disorders (DBDs), both of which are characterized by elevated impulsivity. Participants had to discriminate between two visual gratings, and/or two sounds presented to the right and left ear based on respectively contrast and loudness in a two-alternative forced-choice task. Results show similar task performance across participants. Performance accuracies were highest on audiovisual trials and participants responded fastest on auditory trials, independent of impulsivity levels. Examining the timescale of evidence weighting revealed that early sensory information contributed most to decisions, with dynamic dominance switching between visual and auditory modalities. We found no evidence that the temporal dynamics were affected by impulsivity. These findings suggest that impulsivity and impulsivity related behaviours do not significantly affect MSI during our task and that sensory processing mechanisms remain robust against individual impulsivity levels.

## Introduction

Multisensory integration (MSI) is a process during which information from different sensory modalities is integrated in the brain. MSI allows us to detect and discriminate between sensory events in our environment. As our environment is not static but changes dynamically over time, sensory signals are in addition integrated over time. These integration processes are crucial to create a coherent representation of the world and to effectively adapt to it^1,2^. For example, when you are crossing a busy street, the sounds of vehicles and the changing brightness of headlights have to be integrated correctly. This is vital in order to interpret whether a vehicle is approaching or not and to react accordingly, i.e., to cross the street or not.

MSI often improves perceptual performance, leading to more accurate and efficient decisions compared to unisensory decisions. This phenomenon has been demonstrated by numerous studies in the field of perceptual decision-making^1–7^. Moreover, the field has made considerable progress in understanding how we accumulate sensory evidence over time. A key concept in this context is the sensory weighting profile, which describes how information presented at different time points is weighted when forming a decision. Typically, early information refers to sensory input occurring near the beginning of a stimulus presentation, while late information refers to input closer to the end of stimulus presentation. Studies demonstrated that different strategies for sensory evidence accumulation are used. A number of studies examining the contribution of visual evidence to visual decision-making have shown that observers base decisions more heavily on early information compared to late information^8–13^. Nevertheless, late sensory weighting profiles^14–16^ and flat weighting profiles^7,9^ have also been demonstrated. A recent study revealed that during multisensory decision-making, early visual and early auditory evidence are weighted most heavily^7^. Interestingly, this early weighting was followed by modality dominance switching during which vision dominated first and auditory dominance finalized the decision-making process. These findings highlight that timing and modality of sensory input play a critical role in shaping perceptual decisions.

Impulsivity, defined as a pattern of unplanned actions without regard for the negative consequences that might follow^17^, can result in impulsive decision-making. Impulsivity is a hallmark of several externalizing disorders and is a key bridge symptom of Attention-Deficit/Hyperactivity Disorder (ADHD) and Disruptive Behaviour Disorders (DBDs). ADHD is characterized by inattention, hyperactivity and impulsivity as core symptoms^18,19^. DBDs encompass a group of conditions which are marked by irritable moods and impulsivity^20–22^. There are many studies that have investigated the assessment and treatment of DBDs in clinical practice. However, how MSI is affected in individuals with DBD remains unclear.

The empirical findings on how MSI is affected in adolescents and young adults with ADHD vary considerably and might be related to task complexity. For example, a study using a simple reaction time task found that young adults with ADHD showed similar multisensory benefit compared to neurotypical controls, reflected by faster responses on audiovisual trials compared to unisensory trials^23^. However, when task demands were increased in a follow-up study using a two-alternative forced choice (2-AFC) version of the task, the multisensory benefit disappeared, and individuals with ADHD were overall faster in their responses^24^. Similarly, in a speech-in-noise task, no multisensory benefit was observed when lip movement was added to videos of speaking individuals^25^. More evidence from audiovisual illusions further illustrates this variability. Simple illusionary visual flashes and auditory beeps induce illusions at a similar scale in young adults with ADHD as in controls. However, young adults with ADHD were on average less susceptible to the more complex McGurk illusion^26^. These findings could be attributed to attention problems of individuals with ADHD and may also be related to other traits such as impulsivity. One study that specifically examined the influence of impulsivity on MSI during a Simultaneity Judgement (SJ) task and a Temporal Order Judgement (TOJ) task found that the high impulsivity group had a smaller temporal integration window during MSI compared to the low impulsivity group^27^.

The present study aimed to further investigate the relationship between impulsivity and MSI during a 2-AFC task. We included young adults diagnosed with ADHD and DBD and young adults without these externalizing disorders to establish a range of self-reported impulsivity levels. The task we used was based on the experimental design of a previous study by Tuip and colleagues^7^, during which adolescents had to discriminate between two visual gratings, two sound sources, or a combination of both, based on contrast and loudness. We examined whether individuals with high scores on impulsivity and related behaviours show reduced multisensory benefit compared to individuals with low scores, by manipulating task complexity and measuring both accuracy and reaction times. Based on previous findings that task difficulty can reduce MSI in individuals with ADHD^24,26,29^ and that auditory processing may be selectively affected^28^, we hypothesized a lack or partial lack of multisensory benefit on our task for individuals with high compared to low scores on impulsivity. Specifically, we expected reaction times on audiovisual trials to be similar compared to auditory trials and potentially to visual trials. Performance accuracy on audiovisual trials was expected to be similar compared to visual trials and potentially to auditory trials. In addition, to further understand how MSI would change with impulsivity, we tested whether sensory evidence weighting was altered for participants who scored higher on impulsivity scales compared to participants with lower scores during visual, auditory and audiovisual decision-making.

## Methods

### Participants

This study was approved by The Faculty Ethics Review Board of the Faculty of Social and Behavioural Sciences of the University of Amsterdam. All procedures performed were in accordance with the relevant guidelines and regulations and with the Declaration of Helsinki. Forty-four participants contributed to the study. The sample size was determined based on practical constraints, particularly the feasibility of recruiting participants diagnosed with ADHD or DBDs. Given the limited availability of eligible participants within this population, the final sample size reflects the maximum number that could be recruited during the study period.

Data from four participants were excluded related to technical issues (*n* = 3, ADHD/DBD) or outlier performance (*n* = 1, control), resulting in a final sample of 40 (*M* = 17.2, *SD* = 1, age range = 16–19). Ten participants (eight males and two females) were diagnosed with ADHD or DBD according to the DSM-5^18^ and were recruited via Levvel, an organization specialized in youth mental healthcare. Thirty control participants with no history of ADHD or DBD (22 males and 8 females) were recruited from vocational colleges. Participants were asked whether they had any known visual or auditory impairments, with the exception of corrected-to-normal vision and audition. No participants were excluded based on this criterion. Informed consent was obtained from all participants in accordance with ethical guidelines approved by the institutional ethical committee. Before the experiment, participants received an information letter explaining the study procedures and instructions, including the presentation of visual gratings, sounds through headphones, and combined audiovisual trials. In the consent form, participants confirmed they were 16 years or older and had read and understood the information letter. In addition, they agreed to participate and for their data to be used, and were aware they could withdraw from the study at any time without providing a reason. Participants were naive to the experimental design and study goals at the time of consent. Participants received a monetary reward of 25 euro after participation.

### Questionnaires

At the start of the experiment, participants filled out the Barratt Impulsiveness Scale (BIS-11)^30^ and the Youth Self Report (YSR)^31,32^. The BIS questionnaire is openly available online without any license or copyright restrictions. The YSR was administered under a paid license held by Levvel. The Barratt Impulsiveness Scale (BIS-11) is a self-report questionnaire designed to assess impulsivity through 30 items. We used the Dutch version for the current study^33^. Responses are provided on a 4-point Likert scale, ranging from ‘rarely/never’ (1) to ‘almost always’ (4). Sample items include “I plan tasks carefully,” “I do things without thinking,” and “I am restless at lectures or talks.” In this study, the total BIS-11 score was used. The Youth Self-Report (YSR) consists of 112 items, each rated on a 3-point Likert scale (0 = ‘Not true’, 1 = ‘Somewhat or sometimes true’, and 2 = ‘Very true or often true’). These items are organized into nine problem subscales. The three subscales attention problems (YSR_AT), aggressive behaviour (YSR_AG), and rule breaking behaviour (YSR_RBB) show high concurrent validity for ADHD and DBD^34,35^. Moreover, scores on these subscales were correlated with BIS scores (BIS and YSR_AT: *r*(39) = 0.80, *p* < 0.001; BIS and YSR_AG: *r*(39) = 0.63, *p* < 0.001; BIS and YSR_RBB: *r*(39) = 0.57, *p* < 0.001). Therefore, we included these three subscales as a proxy of impulsive behaviour (i.e., impulsivity related behaviours). Sample items include “I can’t sit still” for the YSR_AT subscale, “I argue a lot” for the YSR_AG subscale, and “I set fires” for the YSR_RBH subscale. The YSR_AT subscale comprises nine items, the YSR_AG subscale 19 items, and the YSR_RBH subscale 11 items. Scores for each subscale were calculated by summing the individual item scores, with higher total scores indicating greater severity of the respective problem.

### Stimuli and procedure

**Fig. 1:**
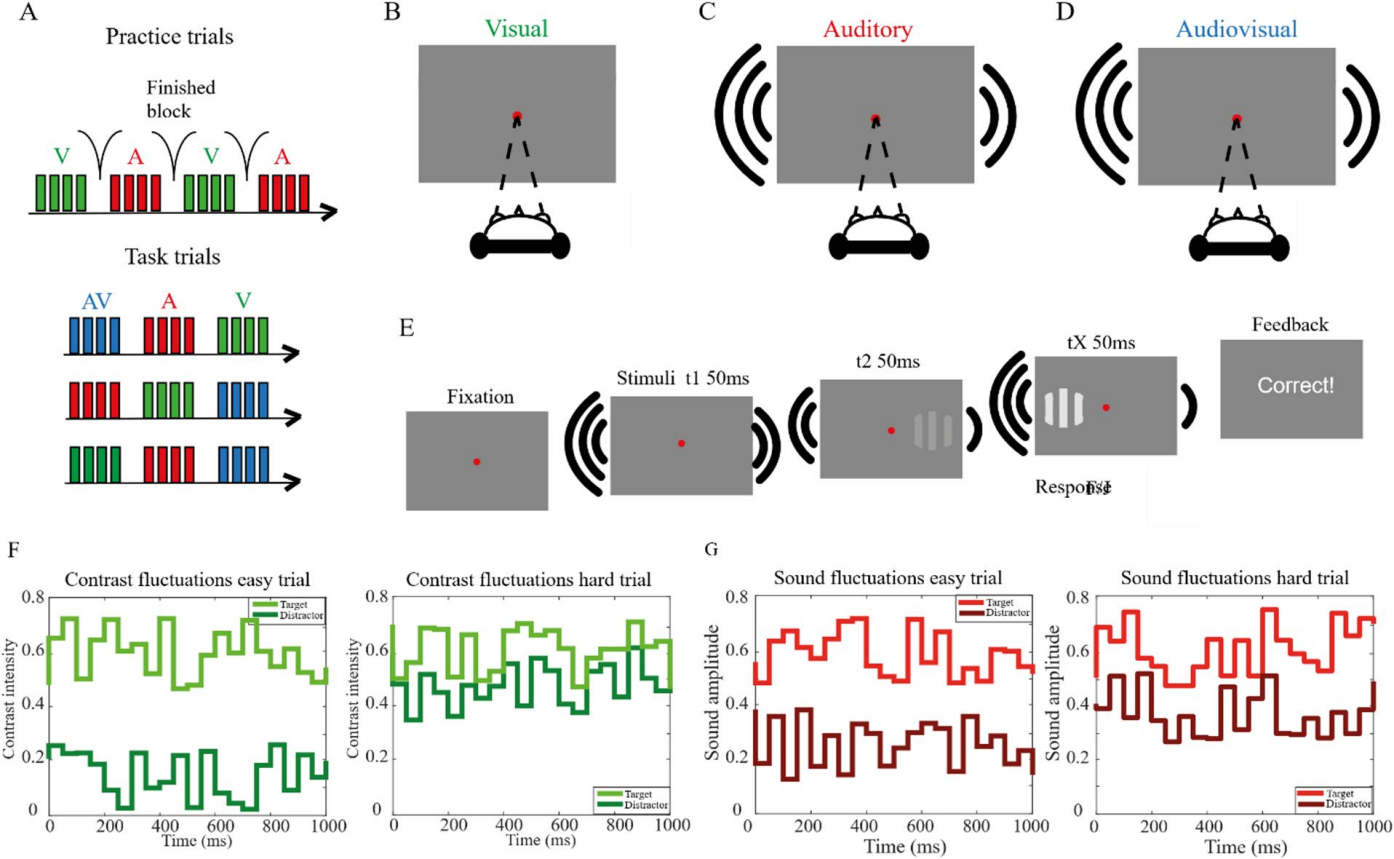
Design of the experimental task. (**A**) The task started with practice blocks of visual and auditory trials after which the task blocks including audiovisual trials were presented. (**B**) On visual trials, participants were asked to indicate whether the left or the right visual grating had the highest contrast (i.e. the target). (**C**) On auditory trials, participants had to press left or right to indicate the loudest sound. (**D**) During audiovisual trials, participants had to indicate the side of the loudest sound and brightest contrast. (**E**) The contrast of the visual stimulus and loudness of the sound fluctuated every 48.6 ms in a random fashion around an average fixed value for the target stimuli. The distractor values differed between the easy and hard trials. Participants had to respond as soon as they perceived the target and received feedback regarding the correctness of their answer after each trial. Stimulus duration was adapted to the average reaction time of each participant represented by time point tX. (**F**) The fluctuations of the target (bright green) and distractor (dark green) contrast on an easy visual trials (left) and hard visual trial (right). (**G**) The fluctuations of the target (bright red) and distractor (dark red) sound on an easy auditory trial (left) and a hard auditory trial (right). Figure adapted from a previous study by Tuip and colleagues^7^.

To measure how modality, difficulty and impulsivity influence MSI, we used a two-alternative forced-choice task similar to the one described by a previous study by Tuip and colleagues^7^. The task was designed with the Psychtoolbox library^36^ in MATLAB R2017a. Figure 1 illustrates the task design. The task consisted of three different modality conditions (visual, auditory and audiovisual) and two different difficulty conditions (easy and hard). There were 612 trials in total divided in 51 blocks of four visual trials, 51 blocks of four auditory trials and 51 blocks of four audiovisual trials. The task started with a block of visual practice trials followed by a block of auditory practice trials, after which visual, auditory and audiovisual blocks were presented in a random order. Trial difficulty (easy or hard) was randomized across trials within each block.

Task presentation and response collection was carried out using a 17.3 inch MSI Bravo gaming laptop with a screen refresh rate of 144 Hz. Participants were centred at a viewing distance of roughly 60 cm from the centre of the gamma-corrected screen. During visual trials, two Gabor gratings with different contrast intensities were shown on the screen on a grey background (Fig. 1B). The centre of the patches were 10 degrees to the left and right from the centre of the screen and had the same random orientation. Participants were instructed to press the ‘f’ key if the stimulus with the highest contrast (i.e., the visual target) was presented on the left side of the screen and the stimulus with the lowest contrast (i.e., the visual distractor) on the right side. The ‘j’ key was the correct response if the visual target was presented on the right side of the screen and the visual distractor on the left side. After each key press participants were provided with response feedback, a grey screen with the text ‘correct!’ or ‘incorrect!’ corresponding with the outcome (Fig. 1E). During auditory trials, two pink noise bursts starting at 7 kHz and linearly decaying to 32 kHz were presented to the left and right ear using headphones with a sampling rate of 44.1 kHz (MD-5000DR, IMG stageline) (Fig. 1C). Participants were asked at which side the sound was highest in amplitude (loudness), i.e., the auditory target, by pressing the same corresponding keyboard keys as during visual trials. During audiovisual trials the auditory and visual stimuli were presented simultaneously and the visual target and auditory target were always on the same side (Fig. 1D, E).

The task included trials of two difficulty levels: easy and hard. The intensity of the distractor stimulus determined the difficulty level while the intensity of the target stimulus was always set at 60%. Throughout the experiment, excluding the practice trials, we used a 1-down-2-up staircase procedure on the hard unimodal trials to determine the visual and auditory distractor baseline intensities for these levels. For the first two trials, the intensity level of the distractor was set at 80% of that of the target. From the third trial on, the distractor intensity decreased with 1% when the trial was incorrect. As a result, the difference between the target and distractor intensity (i.e. the evidence) was increased. When two consecutive trials were correct, the intensity increased with 1% to decrease the evidence. This method resulted in an average of 77.8 ± 5.0% accuracy on hard visual trials, an average of 69.2 ± 8% accuracy on hard auditory trials and an average of 86.6 ± 6.2% accuracy on hard audiovisual trials. We applied a factor of 1.5 to the difference between the target and distractor on the previous hard trials to create the distractor baseline intensity for easy trials. The distractor intensities on audiovisual trials were fixed at the calibrated baseline levels from unimodal trials, allowing for direct comparison and assessment of multisensory benefit.

The stimulus intensities fluctuated every 48.6 ms (seven frames on a 144 Hz monitor) over a baseline value (Fig. 1F, G) to create time-varying contrasts and sounds. We included these fluctuations to retrospectively test which moments in time significantly contributed to the decisions. For the auditory stimulus, the sound level gradually approached the intensity of the next fluctuation over the last 10 ms period to avoid clicks caused by volume transitions. The change in visual contrast was abrupt. The fluctuation range was 14% from baseline intensity resulting in fluctuation intensities between 46% and 74% for the target stimulus. Depending on the task performance and difficulty level, the fluctuations could cause the evidence for the distractor location to be close to or even stronger than evidence for the target location at random points in time (Fig. 1F, G). The motivation for this was to drive participants to evaluate evidence and integrate it over time in order to solve the trial. The majority of the fluctuations, however, were higher on the side of the target. Furthermore, the onset of the fluctuations of the target and the distractor stimuli were simultaneous but the specific intensity values of the fluctuations were calculated randomly. This caused the fluctuations of the target and distractor to be asynchronous at the level of fluctuation direction.

The trial duration of the practice trials was fixed at 2 s. For the following trials, the duration was determined using the reaction times (RTs) of each participant. From the fifth trial up to the 20th trial the RTs of all of the previous trials were averaged. After trial 20, the RTs of the 20 most recent trials were averaged. Subsequently, 65% was added to the average RT value with a lower limit of 500 ms and an upper limit of 2 s to obtain the stimulus length for each trial. Using this method, the trial length felt natural (i.e., under the control of the participant).

### Data analysis

Three participants who were diagnosed with ADHD or DBD experienced technical issues during data collection and were excluded from the analyses. Data of one typically developing control participant was excluded as the median correct reaction time was above two standard deviations relative to the average. The results are based on the data of the remaining 40 participants who all performed above chance level. Due to the low number of participants with ADHD/DBD, a clinical group versus matched control group comparison yielded low statistical power. Instead, we pooled the data of all participants and included the total BIS score and the scores on the YSR subscales attention problems (YSR_AT), aggressive behaviour (YSR_AG) and rule-breaking behaviour (YSR_RBH) as continuous variables in the analyses to test for the effect of impulsivity and impulsivity related behaviours.

A 3 (Modality: Visual, Auditory and Audiovisual) × 2 (Difficulty: Easy and Hard) repeated measures ANOVA (rmANOVA) on the median RTs on correct trials was done to test how modality and difficulty influenced the RTs. This analysis was carried out in JASP^37^. BIS scores and YSR subscale scores as a covariates did not explain the variance significantly and thus we performed Bayesian rmANOVAs^38^ to further examine this lack of significance. To investigate the main effects of Modality and Difficulty, we performed post-hoc tests using the Holm method for multiple comparison corrections.

To examine how Modality, Difficulty and Impulsivity affected performance accuracy we carried out generalized linear mixed-effects models (GLMs)^39^ using JASP. Choice outcomes (correct/incorrect) of all participants on all trials were used to model the probability to make a correct decision with predictors Modality, Difficulty, BIS score and YSR subscale scores:

1 $$\begin{aligned} Y = & \:[1 + {\text{exp}}( - (\beta \:_{0} + \beta \:_{1} *\:Mod_{{ij}} + \beta \:_{2} *\:Diff_{{ij}} \\ & + \beta \:_{3} *\:Imp_{{j,t}} + \beta \:_{4} *\:Mod_{{ij}} *Diff_{{ij}} + b_{j} ))]^{{ - 1}} \\ \end{aligned}$$

Where Y is the response accuracy of the trial (i.e. correct or incorrect), β_0_ is the intercept term and β_1_ reflects the Modality, β_2_ is the Difficulty on trial *i* for participant *j*, β_3_ includes *Imp* which reflects the separate BIS, YSR_AT, YSR_AG or YSR_RBB scores for participant *j* and β_4_ reflects the interaction between Modality and Difficulty. b_j,_ is a random-effects term comprising an intercept and slope for each participant *j* that accounts for potential participant-specific variation in task performance. For post-hoc comparisons of the significant main effects and interaction effect, we tested contrasts with the estimated marginal means using Holm’s *p*-value adjustment. Modality and Difficulty were the only significant predictors and Bayesian GLMs^38^ supported this result. This model was described by the following equation:

2 $$\:Y=\:{[1+\text{e}\text{x}\text{p}(-(\beta\:}_{0}+{\beta\:}_{1}*\:{Mod}_{ij}+{\beta\:}_{2}*\:{Diff}_{ij}+{\beta\:}_{3}*{\:{Mod}_{ij}*Diff}_{ij}+{b}_{j}\left)\right){]}^{-1}$$

Next, we sought to investigate the temporal dynamics of auditory and visual evidence accumulation and how impulsivity influences this. The first step was to pinpoint which moments in time the contrast and sound information contributed to the decisions. We performed different GLMs for the trials of different modalities using MATLAB R2023b (MATLAB function *fitglme* with binomial distribution and logit link). A detailed description of this analysis can be found in a previous study by Tuip and colleagues^7^. To model the probability to make a correct decision on visual trials we used the following equation:

3 $$\:Y=\:{[1+\text{e}\text{x}\text{p}(-(\beta\:}_{0}+{\beta\:}_{1,t}*\:{Vev}_{ij,t}+{b}_{j,t}\left)\right){]}^{-1}$$

Auditory trials:

4 $$\:Y=\:{[1+\text{e}\text{x}\text{p}(-(\beta\:}_{0}+{\beta\:}_{1,t}*\:{Aev}_{ij,t}+{b}_{j,t}\left)\right){]}^{-1}$$

Audiovisual trials:

5 $$\:Y=\:{[1+\text{e}\text{x}\text{p}(-(\beta\:}_{0}+{\beta\:}_{1,t}*\:{Vev}_{ij,t}+{\beta\:}_{2,t}*\:{Aev}_{ij,t}+{b}_{j,t}\left)\right){]}^{-1}$$

*Vev* and *Aev* reflect the evidence of the visual stimulus and auditory stimulus per 48.6 ms time point respectively. The evidence was standardized by z-scoring per time point and trial. The influence of impulsivity and impulsivity related behaviours on the weighting of evidence per time point was assessed by including *Imp* reflecting BIS, YSR_AT, YSR_AG or YSR_RBB scores in the models of the time points where we observed significant coefficient weights. We did this for:

Auditory trials:

6 $$\:Y=\:{[1+\text{e}\text{x}\text{p}(-(\beta\:}_{0}+{\beta\:}_{1,t}*\:{Aev}_{ij,t}+{\beta\:}_{2,t}*\:{Imp}_{j,t}+{b}_{j,t}\left)\right){]}^{-1}$$

Audiovisual trials:

7 $$\:Y=\:{[1+\text{e}\text{x}\text{p}(-(\beta\:}_{0}+{\beta\:}_{1,t}*\:{Vev}_{ij,t}+{\beta\:}_{2,t}*\:{Aev}_{ij,t}+{\beta\:}_{3,t}*\:{Imp}_{j,t}+{b}_{j,t}\left)\right){]}^{-1}$$

Significance of the fixed effects in GLMs 3–7 was tested using the Wald test and corrected for multiple comparisons with the false discovery rate (FDR) method^40^.

## Results

### MSI and impulsivity

We carried out multiple GLMs, described by Eq. 1, to assess how sensory decision-making is affected by modality, difficulty and impulsivity. Participants had an average BIS score of 66.5 ± 10.53, YSR_AT score of 7.9 ± 3.68, YSR_AG score of 6.13 ± 5.09, and YSR_RBB score of 6 ± 3.43. Results from the GLMs revealed no significant effects for the predictors BIS, YSR_AT, YSR_AG and YSR_RBB (all p-values > 0.07). To further examine the absence of an effect of impulsivity and impulsivity related behaviours, we performed Bayesian GLMs to test the evidence in support of the null hypothesis. Table 1 shows the 95% credible intervals of BIS, YSR_AT, YSR_AG and YSR_RBB obtained with these analyses.

**Fig. 2:**
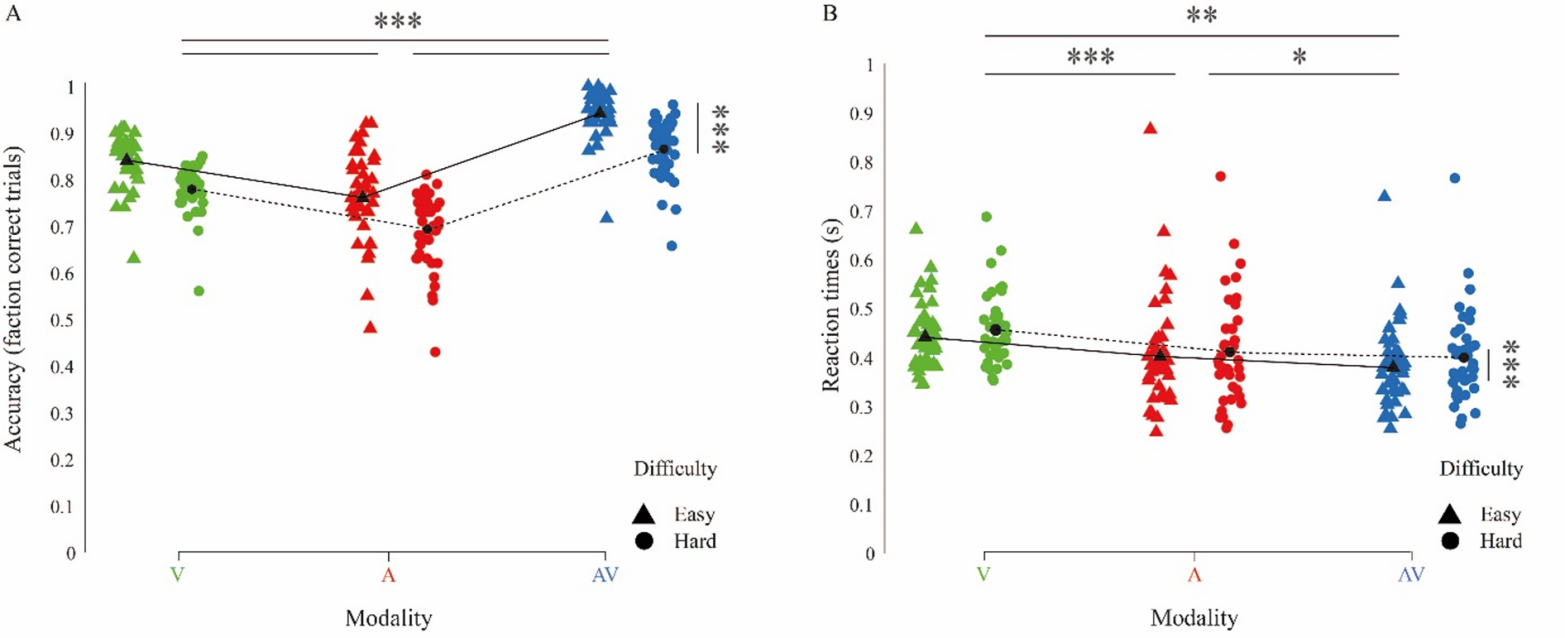
Multisensory benefit on audiovisual trials. (**A**) The performance accuracies of each participant and the averages for visual trials (green), auditory trials (red) and audiovisual trials (blue) for the two difficulty levels (easy: triangle, hard: circle). Performance was significantly higher on audiovisual trials compared to visual trials and auditory trials. Moreover, participants performed significantly higher on visual versus auditory trials and on easy versus hard trials. (**B**) The median correct reaction times of each participant and the average medians reaction times for visual trials (green), auditory trials (red) and audiovisual trials (blue) for the two difficulty levels (easy: triangle, hard: circle). Reaction times were significantly smaller for auditory trials compared to audiovisual and visual trials. Participants responded faster on easy trials compared to hard trials. Significance of the main effects is indicated by solid lines above and next to the plots; *p < 0.05, **p < 0.01, ***p < 0.001).

The 95% credible intervals were centred around zero. It should be noted that credible intervals do not quantify the evidence in favour of the null hypothesis. However, credible intervals centered around zero suggest the data are consistent with the null hypotheses, i.e. that the scores on the various subscales do not influence the decision outcomes during our task. We therefore further analysed the data using a model including only Modality and Difficulty as fixed effects and their interaction term (Eq. 2). The model revealed a main effect of Modality, *Χ*^*2*^ (2) = 72.49, *p* < 0.001 and Difficulty, *Χ*^*2*^ (1) = 82.51, *p* < 0.001 (Fig. 2A). Post hoc tests showed that performance was higher on audiovisual trials (easy 0.968 ± 0.005; hard 0.885 ± 0.009) compared to auditory trials (easy 0.812 ± 0.012; hard 0.74 ± 0.012), *z* = 15.46, *p* < 0.001, and compared to visual trials (easy 0.89 ± 0.006; hard 0.836 ± 0.007), *z* = 12.95, *p* < 0.001. Furthermore, the performance on visual trials was better than on auditory trials, *z* = 8.15, *p* < 0.001. Participants performed better on easy trials compared to hard trials as expected by experimental design. In addition, a significant interaction effect was found between Modality and Difficulty, *Χ*^*2*^ (2) = 41.33, *p* < 0.001. Testing the interaction between Modality and Difficulty showed that on both difficulty levels, performance was better on audiovisual compared to visual and auditory trials (Table 2).

To determine the influence of Modality, Difficulty and Impulsivity on RTs, we performed a set of rmANOVAs including Modality and Difficulty as repeated measures.

**Table 1:** The 95% credible intervals (CI) of the following questionnaire (subscales) predictors in bayesian glms: BIS total score and scores on YSR subscales attention (YSR_AT), aggressive behaviour (YSR_AG) and rule breaking behaviour (YSR_RBB)

Predictor	95% CI	
	Lower	Upper
BIS total	−2.994 × 10^−4^	0.016
YSR_AT	−0.005	0.044
YSR_AG	−0.011	0.021
YSR_RBB	−0.017	0.031

The results show that the predictors were centred around zero and support the null model without these predictors.

factors, and BIS, YSR_AT, YSR_AG and YSR_RBB as covariates. BIS, YSR_AT, YSR_AG and YSR_RBB were not significant covariates (all *p*-values > 0.38) and thus we carried out additional Bayesian rmANOVAs. The data were found to be most likely under a model with a main effect of Modality and Difficulty and an interaction effect between the two terms, *BF10* = 5.92e + 11. Models also including BIS, YSR_AT or YSR_AG were less likely, *BF10* = 4.36e + 11, *BF10* = 5.07e + 11 and respectively *BF10* = 5.17e + 11. A model that included YSR_RBB was slightly more likely than a model without this subscale, *BF10* = 7.21e + 11.

**Table 2:** The results of the post-hoc tests for the effects of modality and difficulty on choice outcome

Modality	Difficulty	
	Easy	Hard
V vs. AV	*z* = 12.92, *p* < 0.001	*z* = 5.40, *p* < 0.001
A vs. AV	*z* = 13.30, *p* < 0.001	*z* = 12.01, *p* < 0.001
V vs. A	*z* = 6.17, *p* < 0.001	*z* = 7.77, *p* < 0.001

Depicted are the modality comparisons per difficulty level, the *z* statistic (z) and the p-values (p) after multiple comparisons correction using the Holm method. The test results show that multisensory improvement was present on both difficulty levels.

Considering the lack of significance for YSR_RBB as a covariate in the conventional rmANOVA and the small increase in Bayes factor in the Bayesian model, we only included Modality, Difficulty and an interaction term for further analyses. The median correct response times of each participant are presented in Fig. 2B. Sphericity was violated (ε = 0.404 for Modality) and therefore Huyn-Feldt corrected results of the 3 (Modality: Auditory, Visual and Audiovisual) × 2 (Difficulty: Easy and Hard) rmANOVA are reported. We found a main effect of Modality, *F*(1.29, 50.37) = 31.58, *p* < 0.001, a main effect of Difficulty, *F*(1, 39 = 35.97, *p* < 0.001) and an interaction between Modality and Difficulty *F*(1.73, 67.39 = 3.86, *p* = 0.03).

Post hoc tests showed that responses to audiovisual trials (easy 383 ms ± 86; hard 401 ms ± 91) were significantly faster compared to auditory trials (easy 405 ms ± 116; hard 411 ms ± 108) *t*(39) = −2.20, *p* = 0.03 and visual trials *t*(39) = −7.67, *p* < 0.001. In addition, participants responded faster to auditory trials compared to visual trials (easy 445 ms ± 69; hard 456 ms ± 72), *t*(39) = −5.47, *p* < 0.001. RTs were faster on easy trials compared to hard trials, *t*(39) = −6, *p* < 0.001. The post hoc results of the interaction effect between Modality and Difficulty revealed that on easy trials, responses were faster on audiovisual trials compared to auditory trials and visual trials. On hard trials, a significant difference in RTs was found for audiovisual versus visual trials (Table 3). Examining the easy versus hard differences revealed a significant faster response in the easy condition for visual trials *t*(39) = −3.33, *p* = 0.06 and audiovisual trials *t*(39) = −5.66, *p* < 0.001.

**Table 3:** The results of the post-hoc tests for the effects of modality and difficulty on reaction times.

Modality	Difficulty	
	Easy	Hard
V vs. AV	*t*(39) = 7.83, *p* < 0.001	*t*(39) = 6.90, *p* < 0.001
A vs. AV	*t*(39) = 2.88, *p* = 0.02	*t*(39) = 1.35, *p* = 0.359
V vs. A	*t*(39) = 4.96, *p* < 0.001	*t*(39) = 5.55, *p* < 0.001

Depicted are the modality comparisons per difficulty level, the degrees of freedom (df), the t statistic (t) and the p-values (p) after multiple comparisons correction using the Holm method. Responses were faster on easy audiovisual trials compared to easy visual and easy auditory trials and that on hard audiovisual trials responses were similar to auditory trials while the significant difference with visual trials holds.

In sum, results from both performance and RTs showed a clear effect of MSI with higher accuracies and faster RTs when both modalities were presented. This effect was not modulated by impulsivity or impulsivity related behaviours. In contrast, Bayesian statistics showed that the data provide strong evidence in favour of the null hypothesis.

### Evidence weighting during visual, auditory and audiovisual decision-making

**Fig. 3:**
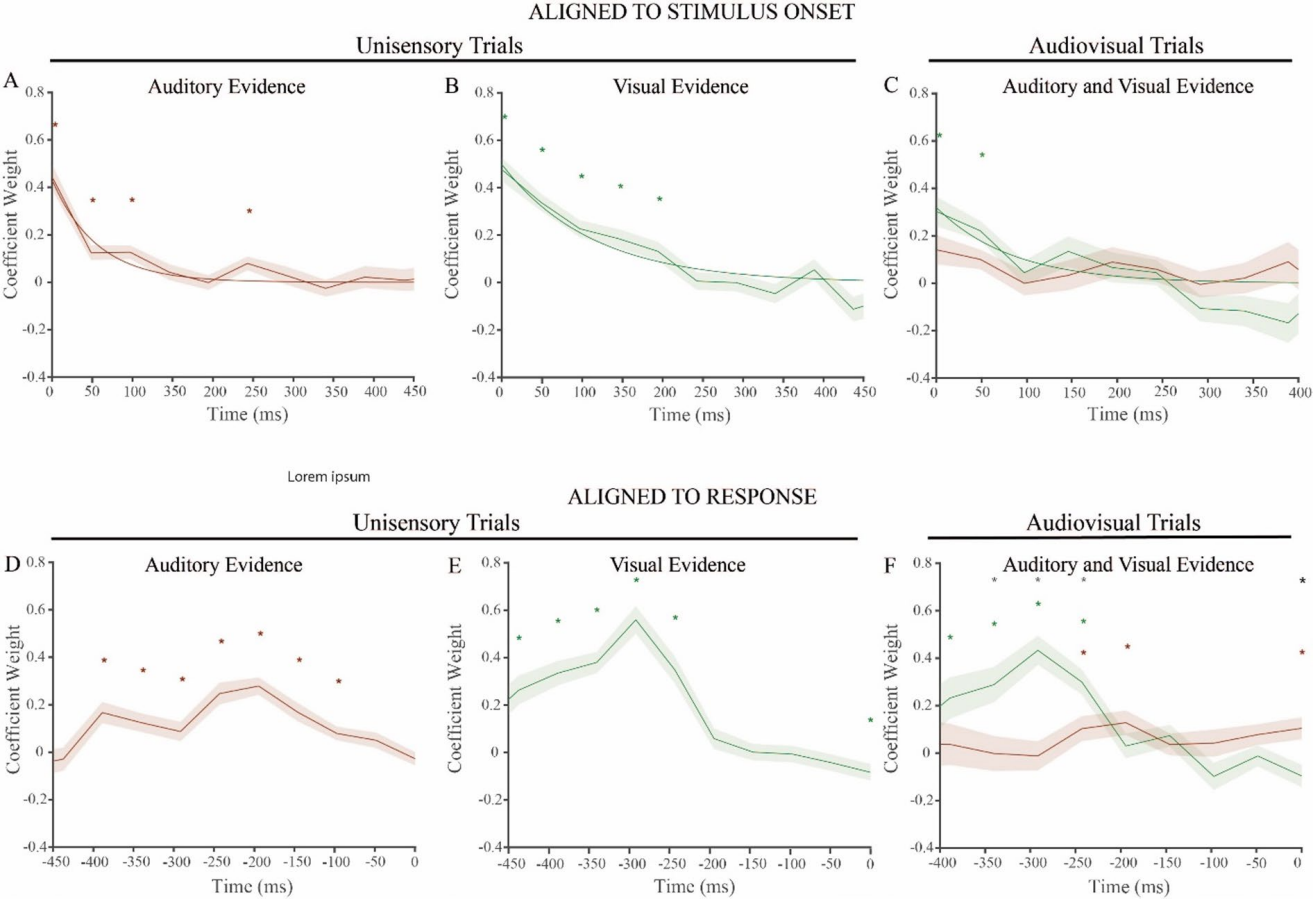
Evidence accumulation difficulties pooled. Weighting profiles A-C are aligned to the stimulus onset and include an exponential fit. Weighting profiles D-F are aligned to the response. (**A**) Early auditory evidence influenced auditory decisions (*n* = 7556 trials) (red asterisks and red exponential fit line). (**B**) Early visual evidence influenced visual decisions (*n* = 7528 trials) (green asterisks and green exponential fit line). (**C**) The first two visual samples contributed to audiovisual decisions with indistinguishable weight from auditory evidence (*n* = 8047 trials) (green asterisks). (D) Auditory evidence influenced auditory decisions until 100 ms before the response (red asterisks). (**E**) Visual evidence influenced visual decisions until 250 ms before the response and the final sample contributed negatively to the decisions (green asterisks). (**F**) In audiovisual trials, visual integration dominated over auditory integration at 350 ms before the response for 150 ms (black asterisks), after which auditory integration occurred around 200 ms (red asterisks). Dominant weighting of the last auditory sample finalized the audiovisual trials (black asterisk).

To further asses the relation between impulsivity and MSI, we next studied audiovisual decision-making at a finer temporal resolution. Even though impulsivity might not affect overall performance, it is possible that the temporal weighing of visual and auditory evidence is modulated by impulsivity. We therefore first performed GLMs per time point (Eqs. 3–5) to generate the weighting plots of auditory evidence on auditory trials, visual evidence on visual trials and auditory and visual evidence on audiovisual trials similar to our previous study^7^. We next included BIS score and YSR subscale scores in the models of the significant time points (Eqs. 6 and 7) to test whether the weighting of sensory evidence at these time points were influenced by impulsivity and impulsivity related behaviours. Figure 3 shows the Beta coefficients per time point up until 75% of the trials were finished. Significant Beta coefficients are marked with an asterisk. We report the statistics of the significant Beta coefficients (*p* < 0.05). In what follows, we first discuss the results of the general weighing profiles before investigating the influence of impulsivity.

The weighting profiles relative to the stimulus onset were characterized by a high contribution of early auditory evidence on auditory trials (*W*-stat range: 7.75–69.06) (Fig. 3A), visual evidence on visual trials (*W*-stat range: 11.04–94.26) (Fig. 3B) and visual evidence on audiovisual trials (*W*-stat range: 24.12–28.91) (Fig. 3C). During audiovisual trials, only the first two time samples of the visual stimulus significantly contributed to the decision. However, the contribution of these two contrast samples were not significantly higher than the contribution of the first two sounds samples. This indicates a lack of modality dominance. To quantify the shape of the sensory weighting profiles, we fitted an exponential distribution to the coefficients of auditory evidence in auditory trials, visual evidence in visual trials, and visual evidence in audiovisual trials. The model statistics and visual inspection revealed that exponential models accurately represented the decline in sensory evidence contribution during this task (auditory weights on auditory trials: *R*^*2*^ = 0.92, *SSE*: 0.01; visual weights on visual trials: *R*^*2*^ = 0.90, *SSE*: 0.04; visual weights on audiovisual trials: *R*^*2*^ = 0.65, *SSE*: 0.07).

Weighting profiles aligned to the stimulus onset captured the influence of the first stimulus samples to the decision. However, samples close to the RTs may be underestimated.

due to the variability in RTs between trials and participants. To address this, we calculated the evidence contribution relative to the response. These weighting profiles are shown in Figs. 3D-F for auditory (*W*-stat range: 0–58.28.28) (Fig. 3D), visual (*W*-stat range: 5.84–95.25) (Fig. 3E), and audiovisual trials (*W*-stat range auditory evidence: 3.25–7.72, *W*-stat range visual evidence: 7.15–50.82) (Fig. 3F). The increasing trend of weights were a consequence of the distribution and underestimation of the first samples when aligning to the response. Focusing on the auditory trials, we observed that participants weight the samples until 100 ms before they respond (Fig. 3D). On visual trials evidence contributed until 250 ms before the response and was finalized by a negative contribution of the last sample (Fig. 3E). Notably, on audiovisual trials (Fig. 3E), a period of visual dominance occurred from 350 until 200 ms relative to the response (*F*-stat: 6.42–26.82) and at 200 ms a period was carried by the weighting of an auditory evidence sample which leaned towards auditory dominance (*F*-stat: 4.05, *p* = 0.09). The final auditory sample was characterized by auditory dominance (*F*-stat: 11.15).

These results reveal that evidence integration throughout the trials is characterized by an exponential decrease in the contribution of evidence. Evidence closer to the response did not contribute to the decision with the exception of the final sample on visual and audiovisual trials. This supports the notion of an overall early weighting profile. Moreover, our results suggest that integration during audiovisual trials can be divided into three distinct epochs. The first epoch involved the weighting of the indistinguishable contribution of both visual and auditory evidence, a pattern most apparent when coefficients were aligned to the stimulus onset. When aligned to the response, the second epoch showed that participants rely solely on visual information until 350 − 200 ms before the response. During the third and last epoch, the final auditory sample was weighted before the response was registered.

### Impulsivity does not affect sensory weighting over time

Next, we included predictors BIS, YSR_AT, YSR_AG and YSR_RBB in the models of the significant time points to test whether the weighting of sensory evidence at these time points were influenced by impulsivity and impulsivity related behaviours. Results from these GLMs revealed no significant effects for the predictors BIS, YSR_AT, YSR_AG and YSR_RBB (all p-values > 0.13). Therefore, we suggest that there is little evidence that BIS score nor YSR_AT, YSR_AG and YSR_RBB scores influenced the weighting of auditory and visual information during this decision-making paradigm.

## Discussion

This study investigated how impulsivity affects MSI during audiovisual decision-making. Our results demonstrate that there is no evidence that performance accuracy and reaction times are influenced by impulsivity in our decision-making paradigm. Multisensory benefit was present regardless of the scores on the impulsivity measure, as well as on behavioural measures related to impulsivity. The temporal weighting analyses revealed that early sensory information exerts most influence on decision-making during this task. Furthermore, enhanced audiovisual decision-making was linked to a sequential modality weighting. Early visual and auditory evidence contributed equally to the decision, followed by visual dominance and finalized by auditory weighting. Impulsivity did not influence these temporal dynamics of sensory integration.

To answer the first question how impulsivity affects general performance during our task, we examined the performance accuracies and reaction times. Impulsivity is a trait that varies across the general population and influences decision-making^41^. In addition, it has been shown that more impulsive individuals exhibit altered time perception and under-reproduction of temporal intervals compared to less impulsive individuals^17^. These differences could influence how sensory information is evaluated over time. Considering impulsivity as a dimensional trait allows for insights into how individual differences, beyond clinical diagnoses, may affect sensory decision-making processes. We found no significant influence of the scores on the impulsivity scale and impulsivity related behaviour subscales on performance accuracy or reaction times suggesting that impulsivity does not affect MSI in our study. ADHD is associated with impulsivity, among a variety of symptoms. Therefore, previous studies on how MSI is affected in individuals with ADHD might provide insights in which mechanisms underlie the influence of impulsivity on MSI. On the level of behaviour, previous studies have shown that those with ADHD respond faster on audiovisual trials compared to visual and auditory trials during a simple reaction time task^23^ and overall faster when task complexity is slightly increased^24^ compared to those without ADHD. Studies by Schulze and colleagues provided more evidence for the relationship between task complexity and MSI. They found that susceptibility for simple sound-induced-flash illusions was not different for adults with ADHD compared to controls whereas the susceptibility for the McGurk effect was lower^26^. Moreover, perceptual load did not influence MSI in adults with ADHD during an auditory flanked visual search task^29^.

Electrophysiological recordings from both humans and monkeys have shown that the parietal cortex plays a central role in MSI as well as attention^42–45^. MSI occurs both in early sensory cortices and at later stages involving parietal and frontal regions, where attentional modulation further refines MSI^46^. Altered MSI in (young) adults with ADHD has been linked to reduced attention by studies showing impaired integration of complex audiovisual stimuli with altered resting-state connectivity in fronto-parietal networks^26^ and reduced parietal and temporal activation during MSI^47^. In contrast, the lack of significant differences in performance related to impulsivity in this study and the similarity of the sensory weighting profiles to those observed in our previous study^7^ might be explained by equal attentional allocation towards the sensory stimuli on all conditions. This in turn could be a result of high overall motivation related to the monetary reward, ensuring consistent engagement and attention throughout the experiment^47^. Another explanation is that the low-level stimuli (i.e. moving gratings and pink noise tones) we used here are low in complexity to a degree that the integration of these properties might occur pre-attentively in a bottom-up fashion^29,48,49^. This idea is supported by the unsuccessful integration of complex stimuli during the McGurk illusion^26^ which might be a result of an enhanced early bottom–up and a suboptimal late top–down MSI in groups with ADHD^29^. We hypothesize that MSI during our task relies mostly on bottom-up processes. In a later processing stage the internal representation of the stimuli might be refined by intact top-down modulation.

The second question we aimed to answer with this study was how visual and auditory evidence is integrated over time and whether this is affected by impulsivity. For this aim, we zoomed into the temporal dynamics of evidence weighting and tested its relationship with impulsivity and impulsivity related behaviours. The weighting profiles revealed early weighting of sensory information on all conditions. Moreover, we observed that more time points contributed to visual decisions compared to auditory decisions, indicating a longer evidence accumulation period during visual decision-making versus auditory decision-making. This is in line with the higher accuracies and slower reaction times on visual trials compared auditory trials. In addition, we observed that audiovisual decision-making was associated with equal contribution of early visual and auditory information after which visual integration dominated followed by the final weighting of auditory information. We found no indication that weighting of visual or auditory evidence was affected by impulsivity or impulsivity related behaviours. Based on these results and the Bayesian statistics that provided evidence that the various impulsivity subscales did not influence the decision outcomes and RTs during our task, we suggest that sensory processing and evidence accumulation mechanisms during our task are immune to individual differences in impulsivity. The weighting profiles mimicked those observed by Tuip and colleagues^7^ which reinforces the reliability and robustness of this finding.

For a comprehensive discussion regarding the factors that influence the shape of weighting profiles and the underlying processes we refer to the discussion of the study by Tuip and colleagues^7^. To summarize, within the framework of signal detection theory (SDT), weighting profiles are thought to reflect an interaction between sensory integration and decision-making processes^9,15,50,51^. Early weighting profiles likely correspond to a bounded accumulation process, where information is integrated until a decision threshold is reached. Early sensory information is weighted heavily while later information has diminished impact once the decision bound has been made. Given the improved accuracy and shorter RTs on audiovisual trials and the observed visual dominance, it is plausible that visual and auditory information streams may be processed separately in early sensory areas after which they are integrated in regions such as the superior colliculus^52^ or higher-order cortical areas^53–55^. The dominance switching that we observed might be driven by dynamically changing connection strengths within and between early sensory areas and frontal and motor regions^56^.

There were some differences in the results that we demonstrated here compared to the study by Tuip and colleagues^7^. First, participants responded faster and more accurate during this study. This difference could be related to the monetary reward participants received here compared to the course credits the students in the previous study received. Our results corroborate findings of other studies that monetary reward can increase speed and accuracy^57,58^. Second, the weighting profiles differed slightly despite the strong similarities. For example, the first auditory evidence sample was not weighted significantly during audiovisual decisions in this study (Fig. 3F). The contribution of the visual evidence was however not significantly higher than the auditory evidence sample, which argues for the contribution of auditory evidence during the first time point. Moreover, the evidence weighting on audiovisual trials was characterized by sequential weighting of visual evidence and auditory evidence. In the previous study, the visual evidence weighting period was reflected by a visual dominance period in both the stimulus aligned and response aligned weighting profiles. The auditory evidence weighting period was reflected by an auditory dominance period right after the visual dominance period in the response aligned weighting profile. In the current study, visual dominance was only observed in the response aligned weighting profile and auditory dominance nearly reached significance (*p* = 0.08) (Fig. 3F). An explanation for this might be that the weighting profiles have been compressed compared to the weighting profiles in the previous study as a result of shorter RTs. This could underlie the less pronounced dominance periods. Another difference is the negative contribution of the visual sample right before the response on visual trials (Fig. 3E) and the positive contribution of the final auditory sample on audiovisual trials (Fig. 3F) in the response aligned profiles. It has been shown that people can initiate an action around from about 200 ms after cue presentation^59^ and can successfully withhold an action if a stop cue is presented 200–290 ms before the action is initiated^60–62^. Therefore, it is unlikely that evidence up to 48,6 ms before the response influenced the choice outcome.

To summarize, our results suggest that general performance on our audiovisual decision-making paradigm was not affected by impulsivity or impulsivity related behaviours. Furthermore, there is no evidence that impulsivity or impulsivity related behaviours influenced the temporal integration of visual and auditory information streams. These results add to a set of mixed results described by a short list of previous studies that investigated MSI in adults with impulsivity associated disorder, ADHD. Our findings corroborate the hypothesis that low complexity stimuli do not engage the sensory processing mechanisms that are disrupted in ADHD.

## Data Availability

The data and MATLAB scripts used for testing and analyses can be accessed on OSF: https://osf.io/3nxuc/.
